# Thyroid carcinoma follow-up: tertiary to primary care transition in Portugal

**DOI:** 10.1530/EC-25-0877

**Published:** 2026-02-19

**Authors:** Renata Barbosa, Susana Garrido, André Couto Carvalho, Cláudia Freitas

**Affiliations:** Division of Endocrinology, Centro Hospitalar e Universitário de Santo António, Unidade Local de Saúde de Santo António, Porto, Portugal

**Keywords:** thyroid neoplasms, thyroglobulin, antithyroglobulin antibodies, patient surveillance, hospital discharge, primary health care, follow-up studies

## Abstract

**Purpose:**

The transition of differentiated thyroid carcinoma (DTC) care from hospital to primary care remains controversial. Current studies suggest that low-risk patients with an excellent response to therapy are safely followed up by primary care professionals. Despite this, discharge practices among Portuguese thyroidologists remain heterogeneous and the success of this clinical transition is yet unknown. This study aims to evaluate primary care compliance to follow-up recommendations for DTC patients after tertiary care discharge.

**Methods:**

A retrospective observational study was conducted including individuals with a history of DTC who were treated in a Portuguese tertiary care hospital and discharged to follow-up at primary care setting, during 2022. Data were collected from electronic records, including thyroglobulin and antithyroglobulin antibody levels.

**Results:**

A total of 134 individuals were discharged. The majority (*n* = 105; 78.4%) were female, with a mean age at discharge of 64 ± 12 years. The most frequent diagnosis was papillary thyroid carcinoma (95.5%, *n* = 128). Regarding treatment, 52.2% (*n* = 70) only underwent thyroidectomy, 44.8% (*n* = 60) underwent thyroidectomy followed by iodine-131 ablation, and 3.0% (*n* = 4) underwent subtotal thyroidectomy. Most DTC cases (86.6%, *n* = 116) were classified as low risk and showed an excellent response to treatment (82.1%, *n* = 110) according to the ATA 2015 Guidelines. One year after discharge, biochemical response evaluated by thyroglobulin and antithyroglobulin levels were registered in 29.1% (*n* = 39) of individuals. Thirteen patients (9.7%) had records of either antithyroglobulin antibodies or thyroglobulin alone.

**Conclusion:**

Patients with low-risk DTC receive suboptimal monitoring after transition to primary care, emphasizing the need to enhance follow-up practices to ensure adequate long-term surveillance.

## Introduction

Differentiated thyroid carcinoma (DTC) is the most common endocrine malignancy, with an increasing incidence, primarily due to the rise in diagnoses of low-risk papillary thyroid cancers (1, 2).

There is limited high-quality evidence regarding the optimal DTC follow-up strategies. Despite its favorable prognosis and low recurrence rates (2–5%), patients typically require long-term follow-up due to its indolent nature, contributing to a significant hospital burden (3).

In addition, studies have shown that the cost of monitoring possible recurrence in patients with low-risk DTC is 6–7 times higher than for those with intermediate- or high-risk DTC (4). Besides this financial hardship, there is also an important social cost, with patients often experiencing anxiety and depression despite the generally favorable prognosis (4, 5).

For all these reasons, identifying the most cost-effective follow-up strategy for this large subset of patients has become a priority (5).

Follow-up protocols vary widely between centers, often influenced by local resources, institutional practices, and practitioner preferences (4).

Most patients with DTC meet remission criteria with an excellent response to treatment, according to the 2015 American Thyroid Association (ATA) Management Guidelines for Adult Patients with Thyroid Nodules and Differentiated Thyroid Cancer (6), which is characterized by a very low risk of recurrence and minimal risk of long-term complications (5). For these low-risk patients, long-term surveillance in primary care may be well suited to take over follow-up care after initial hospital-based management (5, 7).

Evidence suggests that, for these patients, vigilance could be effectively managed in the primary care setting, through plasma thyroglobulin (Tg) and antithyroglobulin antibody (TgAb) monitoring, initiating as soon as five years after first ablation treatment. Late recurrence of DTC in the absence of significant changes in these biomarkers is extremely rare in this population (5).

Recent updates from the 2025 ATA Guidelines have endorsed a more streamlined approach to follow-up in low-risk DTC, recommending cessation of biochemical surveillance after 10–15 years of sustained excellent response. These changes reflect the growing consensus toward reducing unnecessary hospital-based surveillance and shifting care to primary care settings for appropriately selected patients (8).

In this study, we aim to evaluate primary care adherence to follow-up recommendations for low-risk DTC patients with remission status one year after discharge from a tertiary cancer outpatient clinic.

## Materials and methods

We conducted a retrospective observational study at the Unidade Local de Saúde de Santo António, including patients diagnosed with DTC who were discharged from our cancer outpatient clinic to primary care surveillance during 2022. At the time of discharge, patients received a discharge letter addressed to their PCPs (primary care physicians) detailing fully their surveillance recommendations. Patients were only discharged if they had ensured routine primary care follow-up and an assigned PCP.

Demographic and clinical data were collected via electronic health records. Adequate analytical surveillance was defined as the availability of Tg and TgAb levels in the Portuguese Electronic Medical Record (RSE – Registo de Saúde Electrónico) at least one year after hospital discharge. Data were collected during May 2024.

Patients with DTC classified as low or intermediate risk, which had achieved an excellent response to the treatment, regardless of the type of treatment administered, were discharged to primary care after completing at least 5 years of follow-up in the hospital setting. Surveillance recommendations provided to PCPs included thyroid stimulating hormone (TSH) monitoring every 12 months (goal: 0.4–2.0 mU/L) and annual serum Tg and TgAb assessment (6).

This study was conducted in accordance with the Declaration of Helsinki and approved by the local ethics committee (approval number 2025.143[122-CAC/122-CE]).

Statistical analysis was performed using SPSS, version 29.0. Continuous variables were expressed as mean ± standard deviation or median with interquartile range (IQR), while categorical variables were reported as numbers and percentages.

## Results

**Figure 1:**
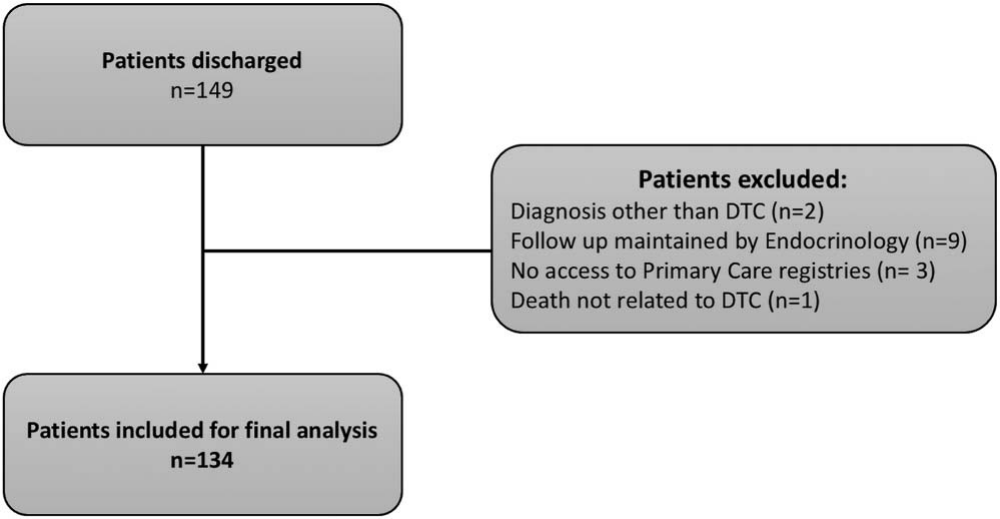
Flow chart illustrating the inclusion and exclusion of patients with differentiated thyroid carcinoma (DTC) discharged from the cancer outpatient clinic to primary care follow-up during 2022.

Of 149 patients discharged in 2022, 134 were included in the final analysis after applying exclusion criteria (Fig. 1). The majority of patients (*n* = 105; 78.4%) were female, and the mean age at discharge was 64 ± 12 years.

The median follow-up duration in our cancer outpatient clinic was 15 (IQR 11) years, with the longest follow-up time of 41 years and the shortest of 5 years. Most patients (85.8%, *n* = 115) had a follow-up period exceeding 10 years.

The most frequent DTC diagnosis was papillary thyroid carcinoma (95.5%, *n* = 128), with 31.3% (*n* = 42) classified as microcarcinoma. The remaining diagnoses included follicular thyroid carcinoma (3.0%, *n* = 4) and oncocytic carcinoma (1.5%, *n* = 2).

Most DTC cases (86.6%, *n* = 116) were classified as low risk according to ATA 2015 Guidelines (6), with the remaining 13.4% (*n* = 18) classified as intermediate risk (Table 1).

**Table 1:** Clinical characteristics of low-risk and intermediate-risk thyroid cancer patients.

	Low risk (*n* = 116)	Intermediate risk (*n* = 18)
Female sex (*n*, %)	92 (87.6)	13 (12.4)
Age at discharge (years, IQR)	66.0 (16)	69.5 (18)
Papillary thyroid carcinoma (*n*, %)	111 (86.7)	17 (13.3)
Median follow-up (years, IQR)	14 (10)	18.5 (14)
Total thyroidectomy (*n*, %)	113 (97.4)	17 (94.4)
^131^I therapy	45 (38.8)	15 (83.3)
Median cumulative ^131^I activity (mCi, IQR)	100 (20)	100 (55)

IQR, interquartile range. Data are expressed as *n* (%) or median (IQR).

Regarding treatment, 52.2% (*n* = 70) underwent only thyroidectomy, 44.8% (*n* = 60) underwent thyroidectomy plus iodine-131 therapy (median cumulative activity of 100 mCi; min–max: 50–400), and 3.0% (*n* = 4) underwent subtotal thyroidectomy.

Clinical responses to treatment were classified as excellent (82.1%, *n* = 110) and indeterminate (17.9%, *n* = 24) according to ATA 2015 Guidelines (6).

**Figure 2:**
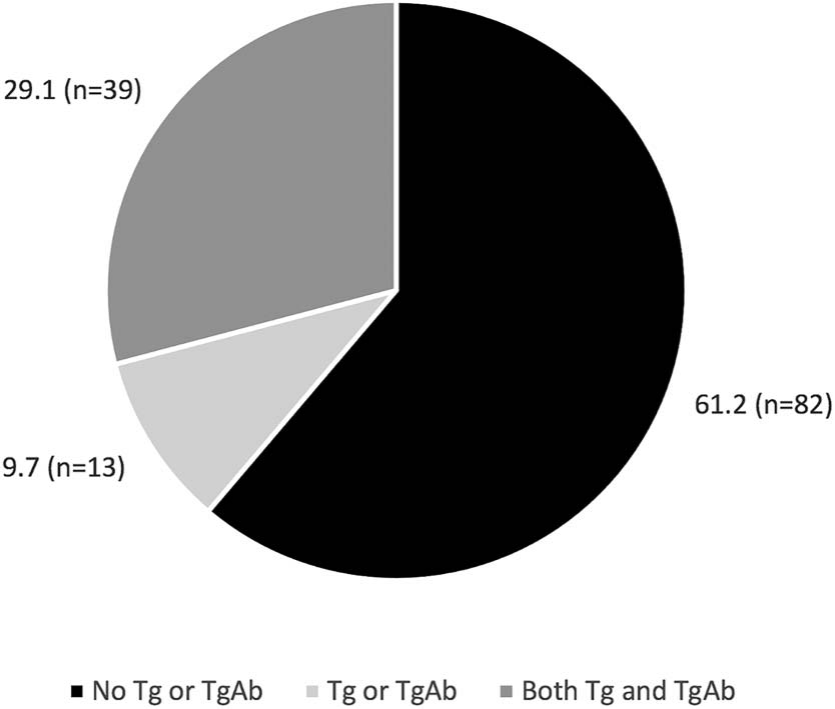
Serum thyroglobulin (Tg) and antithyroglobulin antibody (TgAb) monitoring among DTC patients after transition to primary care. Data are shown as percentage of patients (%).

Since discharged to primary care (median time of 22 months; min–max 16–27 months), Tg and TgAb were recorded in 29.1% (*n* = 39) of individuals. Thirteen patients (9.7%) had records of TgAb or Tg alone. The majority (61.2%, *n* = 82) had no records of both Tg and TgAb levels in the electronic registry platform (Fig. 2). There were no re-referrals to hospital care, and among the 29.1% undergoing fully compliant surveillance, no recurrence cases were observed.

## Discussion

This study found that primary care adherence to surveillance recommendations for patients with DTC following discharge from a tertiary care thyroid clinic was deficient in most cases. To the best of the authors’ knowledge, few studies have addressed this specific aspect of real-world transition from hospital follow-up to primary care in DTC patients.

Despite the safety and somewhat straightforward follow-up considerations of low-risk DTC patients, our findings revealed that, during their first year within primary care responsibility, about two-thirds of these individuals were inadequately followed up.

The long-term surveillance of these low- to intermediate-risk patients, with an excellent response to treatment, currently imposes a significant burden, as they were usually maintained in hospital care for many years, despite their very low recurrence risk.

Previous studies have suggested that it is safe for low-risk DTC patients with an excellent response to treatment to continue their surveillance in a primary care setting five years after treatment, as this approach reduces costs without increasing the risk of recurrence (5, 9).

In accordance with this idea, the 2025 ATA Guidelines now explicitly endorse a more simplified follow-up approach for low-risk DTC patients (recommendation 48) (8). For those treated with total thyroidectomy (with or without iodine-131 therapy) and a sustained excellent response 5–8 years after initial therapy, routine ultrasound can be safely discontinued, and subsequent follow-up may rely solely on periodic biochemical monitoring every 1–2 years. Furthermore, after 10–15 years of stable remission, continued biochemical surveillance is no longer required, as these patients can be considered to have achieved complete remission (8). These updated recommendations reinforce the safety of de-escalating surveillance intensity and support transitioning long-term follow-up to primary care in most DTC cases.

However, significant gaps remain in the literature regarding the role of PCPs during DTC follow-up, and national/regional discharge practices vary widely. There is a lack of consensus among thyroid experts on post-treatment long-term care for low-risk DTC patients, with persisting hospital follow-up being the most common approach (10).

As Haymart *et al.* emphasize, even among thyroid specialists, there remains substantial controversy regarding the optimal extent and duration of surveillance for low-risk DTC, reflecting uncertainty that continues to influence clinical practice (11).

A previous study found that many endocrinologists are reluctant to this transition, even for low-risk or stable patients, often due to concerns about the PCPs’ ability to manage follow-up (12).

Furthermore, PCPs frequently report low confidence in several key aspects of thyroid cancer survivorship care, citing barriers such as limited training, insufficient knowledge, and a lack of clear communication between medical providers (13, 14).

Addressing these challenges requires targeted interventions to enhance PCP involvement in DTC follow-up. These may include continuing medical education programs, improved communication strategies, and clear, standardized discharge protocols that detail follow-up requirements (12, 13). Specifically in our thyroid cancer outpatient clinic, on top of relying on shared electronic records, we send personal follow-up letters directly to PCPs/primary care centers, but even this approach was not enough to ensure a secure transition. Communication between tertiary and primary care teams often remains suboptimal, highlighting the need for structured strategies to support effective coordination and continuity of care. These may include brief, regular case discussions, which could be conducted virtually to allow participation of distant units, and safe, bidirectional communication platforms to provide rapid responses to questions regarding exams or results. Targeted educational initiatives for PCPs, focused specifically on risk-adapted follow-up and guideline-based de-escalation, may help improve confidence and adherence. In addition, clearer and more standardized discharge letters, including concise follow-up algorithms and explicit criteria for re-referral, could further support routine decision-making. Finally, system-level interventions, such as automated electronic reminders or alerts embedded within clinical information systems, could support long-term adherence to follow-up recommendations.

These approaches may help bridge the current gap between hospital care and PCPs, increasing the confidence of endocrinologists/thyroidologists in transitioning patients to primary care and ensuring better long-term surveillance. Strengthening the role of primary care in the long-term follow-up of these patients – particularly those at low risk of recurrence – is a critical step toward achieving more efficient and effective care.

Initiatives such as Canada’s After Cancer Treatment Transition (ACTT) clinic provide a model for an ‘intermediate’ step between hospital specialist and primary care, facilitating continuity of care, improving communication, and preparing both patients and PCPs for a smooth transition, thereby reducing anxiety or lack of confidence (10).

Our findings underscore the urgent need for structured transition programs to ensure seamless coordination between these hospital and primary care providers, enhance patient confidence, and optimize long-term survivorship care outcomes.

The current study has some limitations that should be acknowledged. First, its retrospective nature limits the ability to capture all aspects of follow-up care. This design makes it difficult to associate the outcomes with certain individual characteristics (particularly those that could justify many follow-up failures, such as literacy and social/geographical conditions).

Second, an absence of data on the electronic primary care record platform was interpreted as a lack of compliance to follow-up, although this may not account for potential situations, such as registration errors, incomplete data, or patients opting for follow-up in the private health care. Therefore, the results of this study should be interpreted with some caution, given that its retrospective design and reliance solely on public sector electronic health records may underestimate true overall adherence to follow-up recommendations.

Future studies should adopt a prospective design in order to provide more comprehensive insights into this matter, namely identifying barriers to adequate follow-up and how to improve adherence to surveillance protocols. It would also be interesting to replicate this study in other regions of the country, as the access to primary care in Portugal is very heterogeneous.

In our specific context, a prospective study could involve recalling patients five years post-discharge to systematically evaluate their long-term clinical status, including disease recurrence, relevant biochemical and imaging markers, management of treatment-related complications, and requirement for reintervention. Such data would provide an objective assessment of patient outcomes and inform the real-world consequences of gaps in current follow-up practices.

## Conclusion

This study identified a failure to comply with follow-up recommendations in more than two-thirds of patients discharged from tertiary to primary care. Future research should explore the barriers faced by PCPs, evaluate patient education strategies, and test interventions aimed at improving adherence to follow-up recommendations, ultimately optimizing care pathways for DTC survivors.

## Declaration of interest

The authors declare that there is no conflict of interest that could be perceived as prejudicing the impartiality of the work reported.

## Funding

This work did not receive any specific grant from any funding agency in the public, commercial, or not-for-profit sector.

## Author contribution statement

RB contributed to the conception and design of the study, as well as data acquisition, analysis, and drafting of the manuscript. SG, ACC and CF contributed to data interpretation and critically revised the manuscript. All authors have read and approved the final version of the manuscript.

## Ethics statement

This study was conducted in accordance with the Declaration of Helsinki and approved by the Ethics Committee of Unidade Local de Saúde de Santo António (approval no. 2025.143[122-CAC/122-CE]).
